# Survival According to *BRAF-V600* Tumor Mutations – An Analysis of 437 Patients with Primary Melanoma

**DOI:** 10.1371/journal.pone.0086194

**Published:** 2014-01-24

**Authors:** Diana Meckbach, Jürgen Bauer, Annette Pflugfelder, Friedegund Meier, Christian Busch, Thomas K. Eigentler, David Capper, Andreas von Deimling, Michel Mittelbronn, Sven Perner, Kristian Ikenberg, Markus Hantschke, Petra Büttner, Claus Garbe, Benjamin Weide

**Affiliations:** 1 Division of Dermatooncology, Department of Dermatology, University Medical Center Tübingen, Tübingen, Germany; 2 German Cancer Research Center (DKFZ), Heidelberg, Germany; 3 German Cancer Consortium (DKTK), Heidelberg, Germany; 4 Department of Neuropathology, Ruprecht-Karls-Universität Heidelberg, Heidelberg, Germany; 5 Edinger Institute, Institute of Neurology, Goethe University, Frankfurt, Germany; 6 Department of Prostate Cancer Research, Institute of Pathology, University Hospital of Bonn, Bonn, Germany; 7 Institute of Surgical Pathology, University Hospital Zürich, Zürich, Switzerland; 8 Dermatopathology Friedrichshafen, Friedrichshafen, Germany; 9 Skin Cancer Research Group, School of Public Health, Tropical Medicine and Rehabilitation Sciences, James Cook University, Townsville, Australia; University of Queensland Diamantina Institute, Australia

## Abstract

The prognostic impact of *BRAF-*V600 tumor mutations in stage I/II melanoma patients has not yet been analyzed in detail. We investigated primary tumors of 437 patients diagnosed between 1989 and 2006 by Sanger sequencing. Mutations were detected in 38.7% of patients and were associated with age, histological subtype as well as mitotic rate. The mutational rate was 36.7% in patients with disease-free course and 51.7% in those with subsequent distant metastasis (p = 0.031). No difference in overall survival (p = 0.119) but a trend for worse distant-metastasis-free survival (p = 0.061) was observed in *BRAF* mutant compared to BRAF wild-type patients. Independent prognostic factors for overall survival were tumor thickness, mitotic rate and ulceration. An interesting significant prognostic impact was observed in patients with tumor thickness of 1 mm or less, with the mutation present in 6 of 7 patients dying from melanoma. In conclusion, no significant survival differences were found according to *BRAF*-V600 tumor mutations in patients with primary melanoma but an increasing impact of the mutational status was observed in the subgroup of patients with tumor thickness of 1 mm or less. A potential role of the mutational status as a prognostic factor especially in this subgroup needs to be investigated in larger studies.

## Introduction

The mitogen-activated protein kinase (MAPK) signaling pathway is constitutively activated by *BRAF*-V600 tumor mutations and leads to enhanced mitotic activity [1], [2]. Blocking in *BRAF*-V600 mutant patients by specific inhibitors leads to a high rate of clinical responses and an improved survival of melanoma patients [3]–[5]. Nevertheless, the prognostic relevance of *BRAF* mutations in the natural course of disease is controversial [6]–[20]. A trend towards worse survival of metastatic patients with *BRAF* mutation was found in three patient cohorts [7]–[9]. Similarly, a worse prognosis of metastatic patients with *BRAF* or *NRAS* tumor mutations [10] and of patients with *BRAF* mutant tumors after treatment with temozolomide and bevacizumab [11] was reported before. In contrast, Edlundh-Rose *et al.* did not find any association between the tumor NRAS or *BRAF* genotype and survival in a metastatic setting [12]. Two independent studies reported that a *BRAF* tumor mutation is an unfavorable prognostic factor for stage III patients after resection of loco-regional metastases [13], [14] but others failed to show any negative association with outcome in a similar clinical situation [15]. In non-metastasized patients with primary melanoma, no impact on prognosis was observed thus far in four studies including up to 115 patients [10], [16]–[18]. A recently published meta-analysis of four studies including mainly metastatic patients reported an 1.7-fold increased risk of dying from melanoma for BRAF mutant patients relative to wild-type patients [21].

The aim of the present study was to investigate the prognostic impact of *BRAF*-V600 tumor mutations in patients with non-metastasized cutaneous melanoma after excision of the primary tumor.

## Materials and Methods

### Ethics statement

All patients had given their written informed consent to have their data recorded by the Central Malignant Melanoma Registry (CMMR). This study was approved by the Ethics Committee, University of Tübingen (approval 413/2012BO2).

### Patients

Patients with invasive cutaneous melanoma treated at the University Department of Dermatology in Tübingen, Germany, were identified in the Central Malignant Melanoma Registry (CMMR) database [22]. All patients with initial excision between 1989 and 2006 and available formalin-fixed paraffin-embedded tissue of the primary tumor were included. Data obtained for each patient were gender, age, and the date and cause of death, if applicable. Moreover, time points of initial diagnosis, occurrence of the first distant metastasis, and last follow-up were collected. Histopathologic data of the primary melanoma comprised Breslow's tumor thickness, Clark's level of invasion, ulceration, subtype (superficial spreading melanoma [SSM], nodular melanoma, lentigo maligna melanoma [LMM], acral lentiginous melanoma [ALM]), and mitoses per mm^2^. Only patients with non-metastasized primary cutaneous melanoma at time of initial diagnosis were included (stages I and II).

### Sequencing

Microdissection of formalin-fixed paraffin-embedded tumor tissue was performed to obtain at least 50% tumor cells. After digestion by proteinase K an amplicon containing the *BRAF* codon 600 was amplified by a polymerase-chain-reaction (PCR) assay using forward primer 5′-tcataatgcttgctctgatagga-3′ and reverse primer 5′-ccaaaaatttaatcagtgga-3′. PCR products were analyzed on an agarose gel and purified using USB® ExoSAP-IT® (Affymetrix, Santa Clara, CA). Sanger sequencing was performed in reverse direction and sequences were analyzed with Mutation Surveyor Version 3.20 (SoftGenetics, State College, PA). For all samples which could not be clearly classified as mutant or wild-type, PCR and sequencing was repeated.

### Statistics

The survival times were calculated as follows: Overall survival from the date of the initial diagnosis to the date of last follow-up or death; stage IV survival from the first occurrence of distant metastasis to the date of last follow-up or death; distant metastasis-free survival (DMFS) from the date of the initial diagnosis to the time point of the first occurrence of distant metastasis. Only deaths due to melanoma were considered, whereas patients who died from other cause were censored at the date of death. In three patients who died due to melanoma, the exact date of first occurrence of distant metastases was not available, and the date of distant metastasis was estimated to be 9 months before the melanoma related death, which is the median overall survival time. Estimates of cumulative survival probabilities according to Kaplan-Meier were described together with 95%-confidence intervals and compared using log rank tests. Cox regression analyses were used to determine the independent effects of prognostic factors. All variables were considered in Cox regression analyses and patients with missing data were excluded. Models were established using backward and forward stepwise procedures. Remaining non-significant factors were assessed for potential confounding effects. Changes in the estimates of factors in a model by more than 5% were taken as indicative for confounding. Results of the Cox regression models were described by hazard ratios (HR) together with 95%-confidence intervals, and p-values were based on the Wald test. All Chi square tests were performed 2-sided using Fisher's exact tests. Throughout the analysis, p-values of less than 0.05 were considered statistically significant. All analyses were carried out using SPSS Version 21 (IBM SPSS, Chicago, Illinois, USA).

## Results

### Patients

437 of 451 patients (97%) with successful sequencing were further analyzed. Median age was 57 years (interquartile range 46–67 years) and 52.8% were male. The stage at initial diagnosis according to AJCC was IA in 38.2%, IB in 42.8%, IIA in 12.2%, IIB in 5.1%, IIC in 1.8% of patients, and unknown in two cases because mitotic rate was not available. During follow-up, 58 of 437 patients (12.6%) developed distant metastasis and 52 (11.9%) died from melanoma. Median follow-up was 93 months. None of the patients received treatment with *BRAF* or MEK inhibitors during follow up. A *BRAF*-V600 tumor mutation was detected in 169 patients (38.7%). 150 patients (88.8%) had V600E, 18 (10.6%) V600K, and 1 (0.6%) V600R mutations.

### Clinicopathologic associations according to mutational status

**Figure 1:**
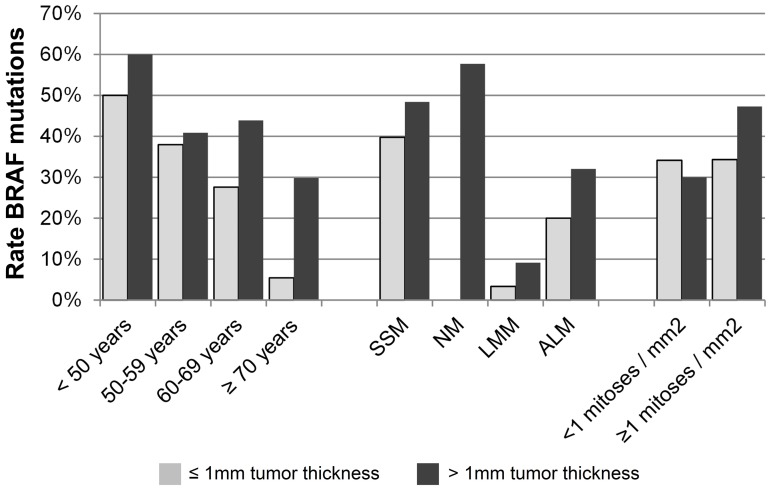
Rate of *BRAF-V600* mutations in patients with tumor thickness of 1 mm or less (grey bars) or more than 1 mm (black bars) according to age (left), histological subtype (middle), and mitotic rate (right). SSM – superficial spreading melanoma; NM – nodular melanoma; LMM – lentigo maligna melanoma; ALM – acral lentiginous melanoma.

Associations between the rate of *BRAF* tumor mutations and demographic, clinical, or histopathologic characteristics are presented in Table 1. The *BRAF* mutational status was strongly associated with age. While the rate of *BRAF* mutant melanoma was 75% in patients younger than 30 years (n = 15) it was only 19% in patients aged 70 years or more (n = 84). This inverse correlation between *BRAF*-V600 mutations and age was stronger among patients with a tumor thickness of 1 mm or less (p<0.001) compared to those with thick primary melanomas (p = 0.034, Figure 1, Table 2). Furthermore, an association with the histological subtype was observed (p<0.001). The majority of nodular melanomas were *BRAF*-V600 mutants (57%); the rate was also high in patients with SSM (43%) but lower in ALMs (30%). In contrast, a *BRAF*-V600 mutation was rarely observed in LMM (6%). The detection of at least 1 mitosis/mm^2^ was associated with mutant *BRAF* in the entire cohort of patients (p = 0.038) and in patients with a tumor thickness of more than 1 mm (p = 0.046) but not in those with thin primary melanomas (p = 1.000, Table 2). No association with tumor *BRAF* mutations was observed for gender, Clark level, ulceration, and tumor thickness. The mutational rate was 36.7% in 379 patients with disease-free course during follow-up and 51.7% in 58 patients with subsequent distant metastasis (p = 0.031).

**Table 1:** Association of *BRAF* mutation status with clinicopathological parameters.

Characteristic	Rate Mutant	Mutant	Wild type	p-value^1^
**All patients**	38.7%	169	268	
**Gender**				0.844
Male	39.3%	90	139	
Female	38.0%	79	129	
**Age**				<0.001
<50 years	53.4%	70	61	
50–59 years	39.3%	42	65	
60–69 years	35.7%	41	74	
≥70 years	19.0%	16	68	
**Ulceration**				0.338
Yes	45.7%	21	25	
No	37.9%	148	243	
**Tumor thickness**				0.190
≤0.50 mm	30.2%	29	67	
0.51–0.75 mm	33.0%	30	61	
0.76–1.00 mm	46.2%	24	28	
1.01–2.00 mm	43.8%	60	77	
2.01–4.00 mm	42.2%	19	26	
>4.00 mm	43.8%	7	9	
**Histological subtype**				<0.001
SSM	43.0%	141	187	
NM	57.7%	15	11	
LMM	5.8%	3	49	
ALM	30.0%	9	21	
**Clark level**				0.277
I–III	36.2%	88	155	
IV or V	41.8%	81	113	
**Mitoses/mm^2^**				0.038
<1	33.2%	72	145	
≥1	43.0%	93	123	

1 p-values are results of Chi-square tests.

**Table 2:** Association of *BRAF* mutational status with clinicopathological parameters stratified according to tumor thickness.

	Tumor thickness ≤1 mm (n = 239)				Tumor thickness >1 mm (n = 198)			
Characteristic	Rate Mutant	Mutant	Wild type	p-value^a^	Rate Mutant	Mutant	Wild type	p-value^a^
**All patients**	34.7%	83	156		43.4%	86	112	
**Gender**				0.419				0.253
Male	32.2%	39	82		47.2%	51	57	
Female	37.3%	44	74		38.9%	35	55	
**Age**				<0.001				0.034
<50 years	50.0%	43	43		60.0%	27	18	
50–59 years	37.9%	22	36		40.8%	20	29	
60–69 years	27.6%	16	42		43.9%	25	32	
≥70 years	5.4%	2	35		29.8%	14	33	
**Ulceration**				0.545				0.605
Yes	0.0%	0	2		47.7%	21	23	
No	35.0%	83	154		42.2%	65	89	
**Tumor thickness**				0.143				1.000
≤0.50 mm	30.2%	29	67					
0.51–0.75 mm	33.0%	30	61					
0.76–1.00 mm	46.2%	24	28					
1.01–2.00 mm					43.8%	60	77	
2.01–4.00 mm					42.2%	19	26	
>4.00 mm					43.8%	7	9	
**Histological subtype**				<0.001				0.001
SSM	39.7%	81	123		48.4%	60	64	
NM		0	0		57.7%	15	11	
LMM	3.3%	1	29		9.1%	2	20	
ALM	20.0%	1	4		32.0%	8	17	
**Clark level**				0.135				0.159
I–III	32.7%	66	136		53.7%	22	19	
IV or V	45.9%	17	20		40.8%	64	94	
**Mitoses/mm^2^**				1.000				0.046
<1	34.1%	57	110		30.0%	15	35	
≥1	34.3%	24	46		47.3%	69	77	

a p-values are results of Chi-square tests.

### Survival Analysis

**Figure 2:**
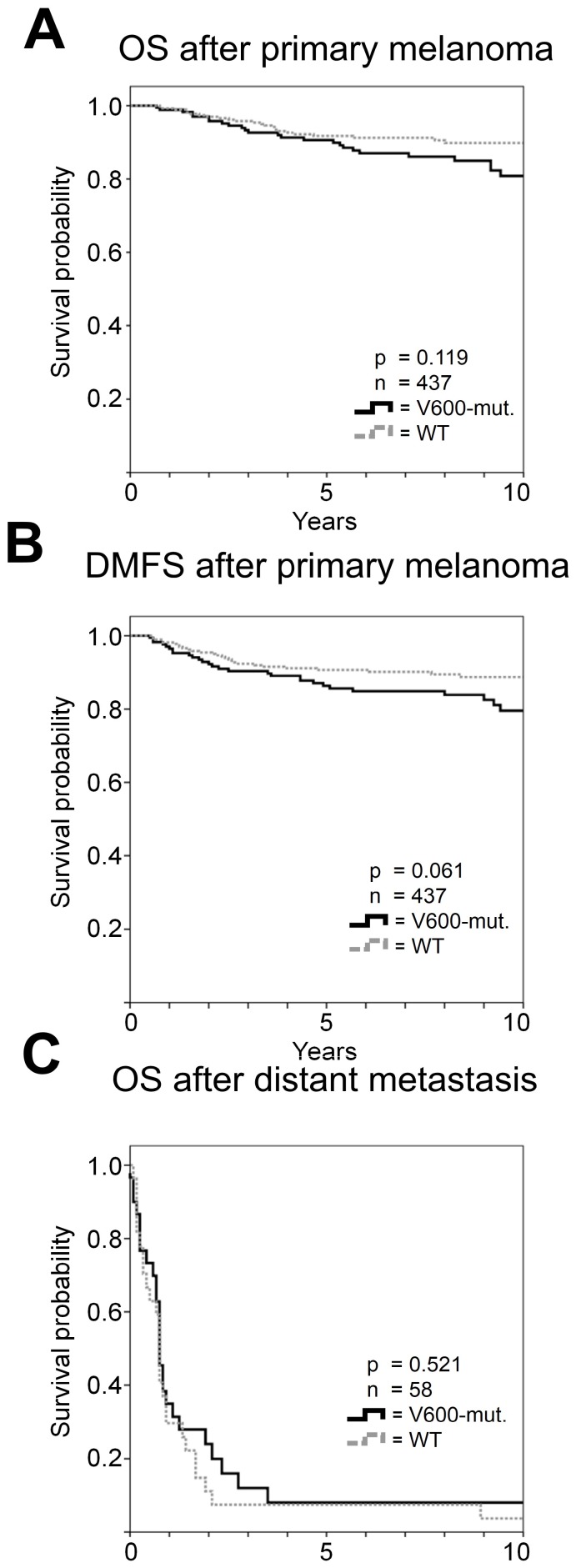
Univariate survival analysis according to *BRAF*-V600 mutational status. No differences in overall survival (A) but a trend for unfavorable distant metastases-free survival (B) was observed in patients with tumor *BRAF* mutations. Survival after occurrence of distant metastasis was not different according to the tumor *BRAF* mutational status (C).

In univariate analysis, tumor thickness, Clark level, ulceration, histopathologic subtype, and mitotic rate were associated with overall survival (all p<0.001). The factors indicating worst prognosis with 10-year survival rates below 50% were a tumor thickness of at least 4 mm (42.9%) and the presence of ulceration (45.1%). In contrast, less than 1 mitosis/mm^2^ and a tumor thickness of 1 mm or less were associated with more than 95% survival probability ten years after initial diagnosis. In Cox regression analysis tumor thickness, ulceration and mitotic rate independently predicted survival (Table 3). A tumor thickness of greater than 4 mm or greater than 2 mm had strongest negative impact on overall survival with a hazard ratio (HR) of 4.7 (p = 0.035) or 4.6 (p = 0.010), respectively, followed by ulceration (HR 3.6, p<0.001) and a rate of at least 1 mitosis/mm^2^ (HR 2.9, p = 0.028). No association of overall survival with the tumor *BRAF*-V600 mutational status was observed (p = 0.119; Figure 2A). No differences in overall survival were detected according to age or gender.

**Table 3:** Analysis of overall survival.

				Univariate analysis			Cox Regression analysis^3^		
Factor	n	%	% Dead	10 Years survival rate (%) [95% CI^1^]		p-value^2^	Hazard Ratio [95%-CI^1^]		p-value
**All patients**	437	100.0	11.9	86.2	[82.5; 89.9]				
**Gender**						0.484			
Male	229	52.4	13.1	85.4	[80.1; 90.7]				
Female	208	47.6	10.6	89.4	[84.9; 93.9]				
**Age**						0.199			
<50 years	131	30.0	9.9	88.4	[81.9; 94.8]				
50–59 years	107	24.5	12.1	86.6	[79.7; 93.5]				
60–69 years	115	26.3	11.3	87.7	[80.8; 94.5]				
≥70 years	84	19.2	15.5	78.2	[62.9; 93.4]				
***BRAF*** **-V600 Mutations**						0.119			
Wildtype	268	61.3	9.7	89.8	[85.9; 93.7]				
V600 Mutation	169	38.7	15.4	80.9	[73.6; 88.2]				
**Ulceration**						<0.001			
Not ulcerated	391	89.5	7.2	91.3	[87.8; 94.8]		1		
Ulcerated	46	10.5	52.2	45.1	[29.8; 60.4]		3.6	[1.9; 6.9]	<0.001
**Histopathologic subtype**						<0.001			
SSM	328	75.2	8.8	89.9	[86.2; 93.6]				
Nodular	26	6.0	42.3	52.3	[30.9; 73.7]				
LMM	52	11.9	5.8	91.5	[81.7; 100.0]				
ALM	30	6.9	26.7	70	[49.0; 91.0]				
Missing data	1								
**Clark level**						<0.001			
Level I–III	243	55.6	5.8	93.3	[89.4; 97.2]				
Level IV–V	194	44.4	19.6	77.5	[70.8; 84.2]				
**Tumor thickness** primary						<0.001			
≤1.00 mm	239	54.7	2.9	95.6	[91.9; 99.3]		1		
1.01–2.00 mm	137	31.4	13.9	84.6	[77.7; 91.5]		1.9	[0.7; 5.4]	0.236
2.01–4.00 mm	45	10.3	40.0	56.6	[40.5; 72.7]		4.6	[1.4; 14.5]	0.010
>4.00 mm	16	3.7	50.0	42.9	[15.3; 70.5]		4.7	[1.1; 19.6]	0.035
**Mitoses/mm** 2						<0.001			
<1	217	50.1	2.8	96.5	[93.3; 99.7]		1		
≥1	216	49.9	20.8	76.5	[69.9; 83.0]		2.9	[1.1; 7.6]	0.028
Missing data	4								

1 95%-CI = 95% confidence interval;

2 p-values are results of log rank tests excluding cases with missing values.

3 Cox Regression analysis was performed in 430 patients.

4 Patients had unknown mitotic rate and in 3 cases censoring occurred before the first event was observed; the model was adjusted for the confounding effects of age, gender, histological subtype, *BRAF*-V600 mutations, and Clark's level of invasion.

There was a trend for unfavorable DMFS in patients with *BRAF* mutant vs. wild-type melanoma (p = 0.061; Figure 2B). 17.8% of patients with *BRAF* mutant tumors but only 10.4% wild-type melanoma patients progressed to stage IV during observation (p = 0.031).

The median overall survival time according to Kaplan Meier after development of distant metastases was 9 months and was not associated with *BRAF* mutational status according to Kaplan-Meier (p = 0.521; Figure 2C). There was no difference in overall survival (p = 0.141) or DMFS (p = 0.251) between 150 patients with V600E mutations compared to 19 patients with V600K or V600R mutations.

### Survival stratified according to tumor thickness

Next, we separately performed the survival analysis for 239 patients with a tumor thickness not exceeding 1 mm and those 198 with tumor thickness larger than 1 mm (Table 4).

**Table 4:** Overall survival stratified according to tumor thickness.

Factor	≤1 mm Tumor thickness								>1 mm Tumor thickness							
	Univariate analysis					Cox Regression analysis^c^			Univariate analysis					Cox Regression analysis^d^		
	n	%	10 Years survival rate (%) [95%-CI^a^]		p-value^b^	Hazard Ratio [95%-CI^a^]		p-value	n	%	10 Years survival rate (%) [95%-CI^a^]		p-value^b^	Hazard Ratio [95%-CI^a^]		p-value
**All patients**	239	100.0	95.6	[91.9; 99.3]					198	100.0	75.1	[68.4; 81.8]				
**Gender**					0.987								0.537			
Male	121	50.6	95.4	[89.9; 100.0]					108	54.5	74.4	[65.4; 83.4]				
Female	118	49.4	95.7	[90.6; 100.0]					90	45.5	75.7	[65.3; 86.1]				
**Age**					0.410								0.300			
<50 years	45	22.7	80.8	[68.7; 92.8]					86	36.0	92.5	[85.0; 99.9]				
50–59 years	49	24.7	71.1	[57.5; 84.6]					58	24.3	na	na				
60–69 years	57	28.8	78.8	[67.4; 90.2]					58	24.3	96.3	[89.2; 100.0]				
≥70 years	47	23.7	64.1	[38.9; 89.3]					37	15.5	96.6	[89.9; 100.0]				
***BRAF-V600*** ** mutations**					0.013								0.994			
Wildtype	156	65.3	99.2	[97.6; 100.0]		1			112	56.6	76.8	[68.2; 85.4]				
V600 Mutation	83	34.7	95.1	[89.4; 100.0]		11.6	[1.2; 111.8]	0.034	86	43.4	73	[62.4; 83.6]				
**Ulceration**					0.926								<0.001			
Not ulcerated	237	99.2	95.6	[91.9; 99.3]					154	77.8	84.5	[77.8; 91.2]		1		
Ulcerated	2	0.8	na						44	22.2	43.3	[28.0; 58.6]		4.2	[2.2; 8.1]	<0.001
**Histological subtype**					0.831								0.015			
SSM	204	85.4	95.6	[91.7; 99.5]					124	62.9	80.7	[73.3; 88.1]				
Nodular	0	0.0	na						26	13.2	52.3	[30.9; 73.7]				
LMM	30	12.6	95.7	[87.3; 100.0]					22	11.2	85.9	[66.5; 105.3]				
ALM	5	2.1	na						25	12.7	63.5	[38.8; 88.2]				
**Clark level**					0.206								0.687			
Level I–III	202	84.5	96.3	[92.6; 100.0]					41	20.7	79.3	[66.6; 92.0]				
Level IV–V	37	15.5	91.8	[80.2; 100.0]					157	79.3	74.2	[66.6; 81.8]				
**Tumor thickness**					0.132								<0.001			
≤0.50 mm	96	40.2	97.4	[93.9; 100.0]												
0.51–0.75 mm	91	38.1	97.2	[91.9; 100.0]												
0.76–1.00 mm	52	21.8	87.5	[73.2; 100.0]												
1.01–2.00 mm									137	69.2	84.6	[77.7; 91.5]		1		
2.01–4.00 mm									45	22.7	56.6	[40.5; 72.7]		2.5	[1.3; 4.9]	0.009
>4.00 mm									16	8.1	42.9	[15.3; 70.5]		2.8	[1.2; 6.5]	0.021
**Mitoses/mm^2^**					0.001								0.021			
<1	167	70.5	98.6	[95.9; 100.0]		1			50	25.5	89.5	[79.3; 99.7]				
≥1	70	29.5	87.6	[76.2; 99.0]		17.9	[1.7; 187.8]	0.016	146	74.5	71.0	[62.9; 79.1]				

a 95%-CI = 95% confidence interval;

b p-values are results of log rank tests excluding cases with missing values.

c 2 patients had unknown mitosis and in 3 cases censoring occurred before the first event was observed; the model was adjusted for the confounding effects of tumor thickness (n = 234).

d 2 patients had unknown mitosis rate and in 1 cases censoring occurred before the first event was observed; the model was adjusted for the confounding effects of mitotic rate and *BRAF-V600* mutations (n = 195).

na = not available.

**Figure 3:**
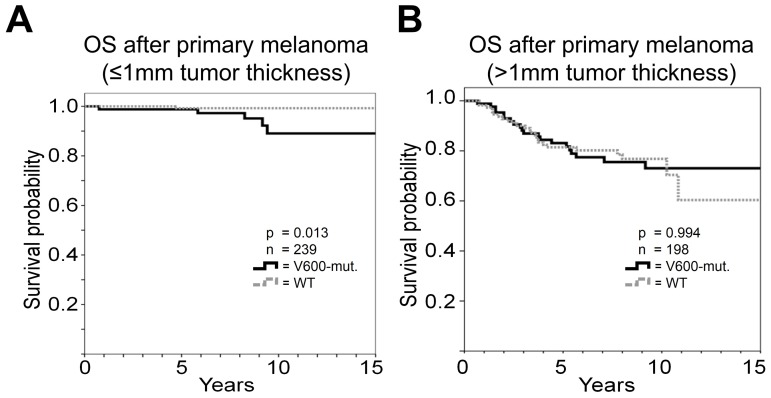
Kaplan-Meier analysis of overall survival (OS) of patients stratified according to tumor thickness for *BRAF-V600* mutant (BRAF-mut.) vs. wild-type (WT) patients with tumor thickness ≤1 mm (A) or with tumor thickness >1 mm (B).

In patients with thin primary melanomas an association with overall survival was observed for the mitotic rate. The 10-year survival rate for patients with less than 1 mitosis/mm^2^ was 98.6% in contrast to 87.6% for the others (p<0.001); this factor had the highest impact in Cox regression analysis (HR 17.9; p = 0.016). The detection of *BRAF* mutations was likewise significantly associated with unfavorable survival (p = 0.013; Figure 3A) and represented an additional independent prognostic factor for melanoma patients with thin primary tumors (HR 11.6; p = 0.034). In patients with thick primary melanoma ulceration, sub-classification according to tumor thickness, rate of mitosis, and histological subtype were associated with survival but only ulceration (HR 4.2; p<0.001) and tumor thickness greater than 2 mm (HR 2.5; p = 0.009) or 4 mm (HR 2.8; p = 0.021) remained independent prognostic factors according to Cox regression analysis. Tumor *BRAF* mutations were not associated with survival (Figure 3B) in these patients with thick primary melanomas.

The difference in DMFS according to the *BRAF* mutational status was also limited to patients with tumor thickness of 1 mm or smaller (p = 0.011) and not evident in those with thicker primary melanomas (p = 0.745).

## Discussion

No prognostic impact of *BRAF*-V600 mutations on overall survival was observed for the entire cohort of 437 non-metastasized melanoma patients in our study. According to Cox regression analysis, we could reproduce all established prognostic factors considered in the AJCC classification with mitotic rate, tumor thickness and ulceration being independently relevant for prognosis of stage I/II patients [23]. A tumor thickness greater than 2 mm or 4 mm (HR 4.6; p = 0.010 or HR 4.7; p = 0.035, respectively) and ulceration (HR = 3.6; p<0.001) were the most important prognostic factors, as already established [24], [25].

Our findings are in agreement with four other studies which investigated the prognostic impact of *BRAF*-V600 tumor mutations in small cohorts of non-metastasized patients and failed to report any relevance of the mutational status [10], [16]–[18].

We observed a higher rate of BRAF mutations in patients with SSM compared to other histopathologic subtypes. This correlation was also found in a meta-analysis which included 36 prior studies and additionally described the localization of the primary melanoma in non-chronically sun-damaged skin as a factor associated with a high rate of BRAF mutations [26]. In our study, we did not include data on early-life UV-exposure, which was reported to correlate with the BRAF mutational rate [27], but a higher rate of mutations in young patients independent of UV-exposure was observed by us, as well as by others [7], [8].

In contrast to non-metastasized patients, the prognostic relevance of *BRAF* mutations has been reported previously for patients with distant metastasis [7], [8], [11]. In the study of Long *et al.*, overall survival after development of distant metastasis was reduced in *BRAF* mutant patients, while there was no difference in DMFS [7]. In contrast, in our study we observed no difference in stage IV survival according to the mutational status but a strong trend (p = 0.061) for an impaired DMFS in *BRAF* mutant patients. Similar results were reported by Edlundh-Rose *et al.* who analyzed 214 metastasized patients [12].

**Figure 4:**
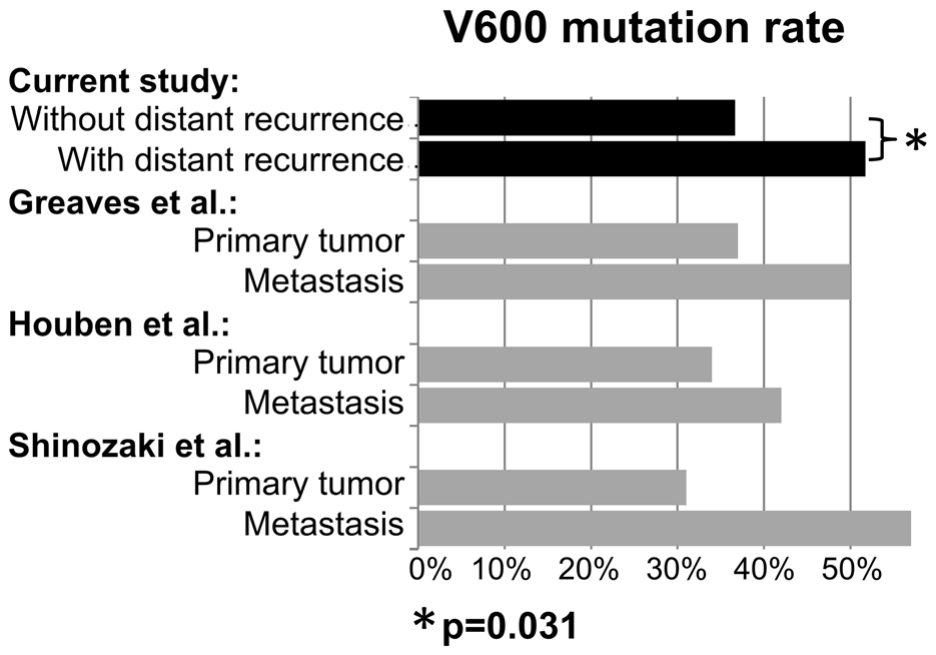
Rate of *BRAF-*V600 tumor mutations according to disease outcome. A significantly lower rate of *BRAF*-V600 tumor mutations was observed in 382 patients who did not develop distant metastasis during follow-up (upper black solid bar - w/o distant metastasis) compared to 55 stage I/II patients who had distant recurrences in the further course of disease (black solid bar - with distant metastases) in our study (36.6% versus 52.7%; p = 0.026). A similar difference in mutational rate was reported in prior studies comparing the mutational rate in metastases of late-stage melanoma patients and primary tumors of stage I/II patients.

In addition to differences in DMFS, the higher mutational rate in 58 stage I/II patients who developed distant metastases during follow-up compared to 379 patients without subsequent distant recurrence provides further evidence that a *BRAF*-V600 mutation may indicate an increased risk of developing distant metastasis (51.7% versus 36.7%; p = 0.031). Lower rates of *BRAF*-V600 mutations had also been previously reported after analysis of primary tumors compared to metastasis (Figure 4) but was explained by the acquisition and accumulation of *BRAF* mutant tumor cells during the course of disease [10], [17], [28]. This explanation is in contrast to recent publications reporting a high proportion of patients with consistent mutation patterns when comparing pairs of primary tumors and metastases of the same individuals [29], [30] and the differences in the rate of *BRAF* mutations in primary tumors if stratified according to disease outcome in the current study.

The conflicting results for DMFS can also be explained by patient selection in prior studies, which limited the analysis of DMFS to patients, who had already developed distant metastasis [7]. In the current study, which represents the largest analysis of the prognostic impact of *BRAF* mutations in non-metastasized melanoma patients thus far, we retrospectively analyzed the *BRAF* status in patients who had not been selected on the basis of their later disease course or outcome. The strong trend for a worse DMFS in *BRAF* mutant patients observed in our cohort is completely lost, if the analysis is restricted to patients who developed distant metastasis in their later course of disease (data not shown).

The conflicting results for stage IV survival might also be explained by a potential patient selection bias. In some prior retrospective prognostic studies using already available institutional data from mutational testing it has to be assumed that the BRAF V600 status was tested due to the intention to treat with a *BRAF*- or *MEK* inhibitor at least in a subset of patients (e.g. [9]). But in order to analyze the treatment-unrelated “natural” impact of *BRAF*-V600 tumor mutations only patients with confirmed *BRAF*-mutations who finally did not receive subsequent inhibitor treatment can be considered. Reasons among others for non-treatment with inhibitors in *BRAF*-V600 mutant patients could be exclusion criteria in the frame of clinical studies (e.g. elevated LDH or occurrence of brain metastases), decrease of performance status or early death due to disease progression. Therefore these patients might represent a cohort biased towards worse prognosis.

We included a majority of patients (n = 239) with tumor thickness of 1 mm or less. In the 7^th^ edition of the AJCC staging classification, ulceration and mitotic rate are considered for classification purposes in these patients [31]. Even if prognosis is generally considered good, between 5% and 10% eventually die from melanoma [31]. Additional prognostic markers are therefore desirable for this large subgroup, representing more than 40% of all stage I/II patients [31]. We showed that *BRAF*-V600 mutations in melanoma cells represent a prognostic factor indicating worse distant metastasis-free and overall survival of non-metastasized patients with a tumor thickness of 1 mm or less. These are results of a subgroup analysis and have to be interpreted with caution, as it is based on a small number of events. On the other hand the present study is the first which focused on low risk patients. Only Shinozaki *et al.* [17] included a limited number of patients (n = 19) with a tumor thickness of less than 1 mm, and a selection towards thick primary melanomas was likewise evident in other previous studies performed in non-metastatic patients. Our results may provide a rationale to analyze the prognostic impact of *BRAF* mutations in non-metastasized low risk patients in larger studies.

In conclusion, no significant survival differences were found according to *BRAF*-V600 tumor mutations in patients with primary melanoma but an increasing impact of the mutational status was observed in the subgroup of patients with tumor thickness of 1 mm or less. A potential role of the mutational status as a prognostic factor especially in this subgroup needs to be investigated in larger studies.
